# Raman spectroscopic fingerprinting uncovers a multi-scale structural–mechanical–transcriptomic coupling landscape in osteoporosis

**DOI:** 10.3389/fendo.2026.1860651

**Published:** 2026-05-28

**Authors:** Jinyang Wang, Yongxi Lu, Xinwei Zhou, Lei Huang, Xuanyi Li, Xiaoxing Kou, Yang Cao, Yang Yang

**Affiliations:** 1 Hospital of Stomatology, Guanghua School of Stomatology, Sun Yat-sen University, Guangzhou, China; 2 Guangdong Provincial Key Laboratory of Stomatology, Guangzhou, China; 3 South China Center of Craniofacial Stem Cell Research, Guanghua School of Stomatology, Sun Yat-sen University, Guangzhou, China

**Keywords:** bone composition, osteoporosis, Raman spectroscopy, single-cell RNA sequencing, trabecular bone

## Abstract

**Background:**

Osteoporosis is a systemic skeletal disorder characterized by reduced bone strength and increased fracture risk. Conventional evaluation relies mainly on bone mineral density and microarchitecture, but these measures do not fully capture the tissue-level material properties that contribute to fragility. Here, we integrated Raman-derived compositional information with microarchitectural, local mechanical, and single-cell transcriptomic data to map a multi-scale coupling landscape and identify a conserved compositional fingerprint of osteoporotic trabecular bone.

**Methods:**

Trabecular bone alterations were profiled by Raman spectroscopy in murine models of natural aging and ovariectomy (OVX)-induced osteoporosis. A linear support vector machine (LSVM) classifier was trained for automated phenotyping of Raman spectra. Raman-defined spectral features were then integrated with micro-CT-based microarchitectural measurements, nanoindentation-derived local mechanical properties, and single-cell RNA sequencing (scRNA-seq) of bone marrow mesenchymal stem cells (BMMSCs) to contextualize compositional changes across structure, mechanics, and remodeling programs.

**Results:**

We identified a conserved osteoporotic Raman fingerprint characterized by reduced phosphate and collagen signals and increased lipid-associated bands. These compositional signatures were strongly associated with micro-CT-defined structural deterioration and nanoindentation-derived local mechanical alterations, specifically reduced hardness and increased elastic modulus. Furthermore, scRNA-seq revealed shifts in BMMSC transcriptomic programs related to mineral, extracellular matrix, and lipid metabolism that paralleled the Raman-defined changes. The OVX model further confirmed the etiological robustness of this Raman fingerprint in capturing multi-scale alterations in bone quality.

**Conclusions:**

Raman-based compositional fingerprinting provides a multidimensional readout that can be integrated with structural imaging, mechanical testing, and transcriptomic profiling. This cross-scale framework refines osteoporosis evaluation, supports the development of advanced diagnostic strategies, and offers mechanistic insight into bone fragility beyond conventional structural metrics.

## 1 Introduction

Osteoporosis is a systemic skeletal disorder characterized by increased fracture susceptibility in aging populations and imposes a substantial global clinical and socioeconomic burden (1). Current diagnostic approaches rely primarily on bone mineral density and trabecular microarchitecture, yet these structural metrics explain only part of fracture-risk variation (2). This discrepancy underscores the important contribution of tissue-level material properties to bone fragility, which remains insufficiently captured by standard assessments.

Bone is a hierarchical mineralized tissue whose mechanical competence depends on the coordinated interplay between its inorganic and organic phases (3). Physicochemical features such as mineral crystallinity, collagen integrity, and lipid accumulation shape tissue quality and ultimately influence skeletal fragility (4, 5). Because compositional alterations in trabecular bone may arise before overt architectural deterioration, conventional imaging lacks the chemical sensitivity needed to detect these early molecular changes (6). In addition, structural imaging, mechanical testing, and molecular profiling are typically performed as separate assays, making it difficult to integrate chemistry, mechanics, and cellular programs at matched spatial scales or to understand how biological remodeling translates into loss of mechanical competence.

Raman spectroscopy offers a compelling analytical alternative. As a label-free and non-destructive vibrational technique, it provides spatially resolved molecular fingerprints of multiple bone constituents within a single acquisition (7). Although Raman-based compositional profiling has increasingly been used to interrogate bone chemistry, its value as a quantitative and integrative readout for osteoporosis remains underdeveloped (8, 9). Most studies have focused on selected spectral features or pairwise correlations, leaving a major gap in full-spectrum, cross-scale integration with microarchitecture, local mechanics, and transcriptomic remodeling measured in parallel and in regionally matched samples (10, 11). Addressing this gap could position Raman spectroscopy as a multidimensional compositional readout that complements conventional structural metrics and enables more mechanistic interpretation of bone fragility.

In this study, we integrated Raman-derived compositional data with structural, mechanical, and transcriptomic datasets to construct a multi-scale coupling landscape and delineate a conserved compositional fingerprint of osteoporotic trabecular bone. We applied Raman spectroscopic fingerprinting to characterize compositional differences between trabecular bone from young and aged mice and developed a machine-learning model to classify these spectral phenotypes. We then linked the Raman-defined signatures to micro-CT-quantified microarchitectural deterioration, nanoindentation-derived local mechanical alterations, and pathway-level changes in bone marrow mesenchymal stem cells (BMMSCs) identified by single-cell RNA sequencing (scRNA-seq), enabling region-consistent association of composition with structure, mechanics, and cellular programs. After validating these signatures in an ovariectomy-induced osteoporosis model, we showed that this multimodal strategy captures coordinated remodeling across molecular, structural, and functional dimensions. Together, these findings highlight Raman spectroscopy as a data-rich modality with translational potential for advanced bone-quality evaluation and diagnostic development.

## 2 Materials and methods

### 2.1 Animals and ethical approval

Young (2 months old) and aged (18 months old) male C57BL/6J mice were obtained from GemPharmatech (China). All experimental procedures were approved by the Institutional Animal Care and Use Committee of Sun Yat-sen University (approval no. SYSUIACUC-2025-002056) and were performed in strict compliance with institutional guidelines for animal welfare.

### 2.2 Ovariectomized mouse model

Female C57BL/6J mice at 8 weeks of age were randomly assigned to an OVX group or a sham-operated control group. Bilateral ovariectomy was performed under general anesthesia. For the surgical procedure, mice were placed in a prone position, and a small dorsal incision was made to access the retroperitoneal space. The ovaries were identified and excised in the OVX group, followed by wound closure with sutures. Sham-operated mice underwent identical surgical exposure without ovary excision. All animals were sacrificed four weeks postoperatively for subsequent analyses.

### 2.3 Micro-computed tomography analysis and sample preparation

After sacrifice, femora were harvested and cleared of residual soft tissue. Specimens were fixed in 4% paraformaldehyde for 48 h. Micro-CT was performed using a high-resolution system (Scanco Medical AG, Switzerland) with an isotropic voxel size of 20 µm, an X-ray tube voltage of 70 kVp, and a tube current of 200 µA. Three-dimensional images were reconstructed and analyzed using the manufacturer’s software and Amira software (Visage Imaging, Germany) to quantify bone morphometric parameters.

### 2.4 Nanoindentation testing

Femoral specimens were dehydrated through a graded ethanol series, embedded, sectioned, and ground to obtain a flat and smooth surface suitable for indentation. Nanoindentation was performed within trabecular regions using a nanoindenter (Keysight G200, USA) to measure local hardness and elastic modulus. The indentation protocol was displacement-controlled at a rate of 10 nm/s until a fixed depth of 2000 nm was reached, corresponding to approximately 80 mN, followed by a 10-s hold at peak load. Mechanical parameters were derived from the load–displacement curves using the Oliver–Pharr method.

### 2.5 Histological staining and imaging

After decalcification in EDTA, femoral specimens were paraffin-embedded and sectioned serially. Sections were stained with hematoxylin and eosin according to standard protocols, and whole-slide images were acquired using an Aperio AT2 scanning system (Leica, Germany).

### 2.6 Raman spectroscopy and Raman imaging

Raman spectroscopy was performed on sections obtained from the same samples used for histological staining. Before Raman acquisition, paraffin-embedded sections were deparaffinized and thoroughly rinsed with ultrapure water to remove residual solvents and minimize background interference. Raman spectra and images were acquired using a WITec 300R Raman microscope (WITec, Germany) equipped with a 532 nm laser. Laser power at the sample surface was maintained between 5–15 mW depending on calibration. The spectra, covering 400–3300 cm^-1^, were collected with 0.1 s exposure per point and a mapping step size of 500 nm. Raman mapping was performed over representative regions of interest (ROI) in femoral sections using a consistent scan area to generate chemical distribution maps. For downstream analyses, approximately 100 individual spectra originating from trabecular bone were randomly extracted from each mapping dataset. In addition, trabecular spectra were averaged to obtain a representative mean spectrum for each experimental group. Spectral smoothing was applied when necessary.

### 2.7 Raman spectral preprocessing and feature extraction

Raman datasets were preprocessed using a multi-step workflow. Spectra deemed unreliable were removed at the initial stage, particularly those affected by laser-related degradation or measurement artifacts, as indicated by unusually high baseline levels, detector overexposure, loss of diagnostic Raman features, or markedly poor signal-to-noise performance. Cosmic-ray spikes were then identified and removed, and autofluorescence background was corrected by baseline modeling and subtraction. To reduce intensity variability across measurements while preserving relative compositional features, each spectrum was normalized by its total integrated area over the full spectral range such that the summed intensity equaled unity.

### 2.8 ScRNA-seq and analysis

Bone marrow samples were collected from young and aged mice (n = 3 per age group), and single-cell suspensions were processed for scRNA-seq library construction using the DNBelab C Series kit (MGI). Individual cells were encapsulated in gel bead-in-emulsion (GEM) droplets, followed by cell lysis and reverse transcription to generate uniquely barcoded cDNA; cDNA was then PCR-amplified, fragmented to an average size of 200–300 bp, and subjected to end repair, A-tailing, and adaptor ligation. Indexed libraries were generated by additional PCR amplification and run on a DNBSEQ-T7 instrument using paired-end sequencing with 41 bp for read 1 and 100 bp for read 2. Sequencing data were filtered to remove adaptor sequences and low-quality bases using fastp. Clean reads were mapped to the mm10 mouse reference genome with STAR, followed by generation of a UMI count matrix in a cell-by-gene format. Downstream analyses were performed in R using Seurat (version 5.0), including data normalization, selection of highly variable genes, and feature scaling, and principal component analysis was used for dimensionality reduction, batch effects were corrected with Harmony, clustering was conducted using the FindNeighbors and FindClusters functions, and cell identities were assigned based on canonical marker genes curated from the CellMarker 2.0 database.

### 2.9 Linear support vector machine-based modeling of Raman spectra

For analysis, Raman spectra were restricted to the 400–1800 cm^-1^ and 2700–3300 cm^-1^ ranges, corresponding to the fingerprint and high-wavenumber regions, which capture key biochemical signals of bone tissue, while the 1800–2700 cm^-1^ region that considered a silent region was excluded due to minimal information and high background noise. Spectra were smoothed using a Savitzky–Golay (SG) filter with a third-order polynomial and a window length of 15 points, followed by baseline correction using asymmetric least squares (ALS) with λ = 1 × 10^6^, *p* = 0.001, and 10 iterations, and normalized by standard normal variate (SNV) transformation. Principal Component Analysis (PCA) was applied to the selected spectral regions to extract orthogonal components explaining 80% of the variance, summarizing global biochemical changes while reducing multicollinearity and overfitting. Classification was performed using a LSVM, suitable for high-dimensional spectral inputs with limited samples. Model performance was assessed using leave-one-group-out (LOGO) cross-validation (CV), in which all spectra from one mouse were withheld as an independent test set to prevent information leakage. Within each outer fold, hyperparameter selection was performed exclusively on the training data using Group K-fold CV with balanced accuracy as the optimization criterion to determine the optimal regularization parameter. After training on the fold-specific training set, the model was evaluated on the held-out mouse, and overall performance was summarized using accuracy, sensitivity, specificity, and receiver operating characteristic (ROC) curves.

### 2.10 Statistics

Statistical analyses were performed using GraphPad Prism 10.0, and data are presented as mean ± SD. Normality was assessed prior to statistical testing. For analyses involving more than two groups, one-way ANOVA was performed followed by *post hoc* pairwise comparisons with Bonferroni correction to adjust for multiple testing. Pearson correlation analyses were performed to assess linear associations between variables. Sample sizes and definitions of replicates are provided in the figure legends. A *p* value < 0.05 was considered statistically significant.

## 3 Results

### 3.1 Raman spectroscopic imaging identifies an *in situ* compositional fingerprint of osteoporotic trabecular bone

**Figure 1:**
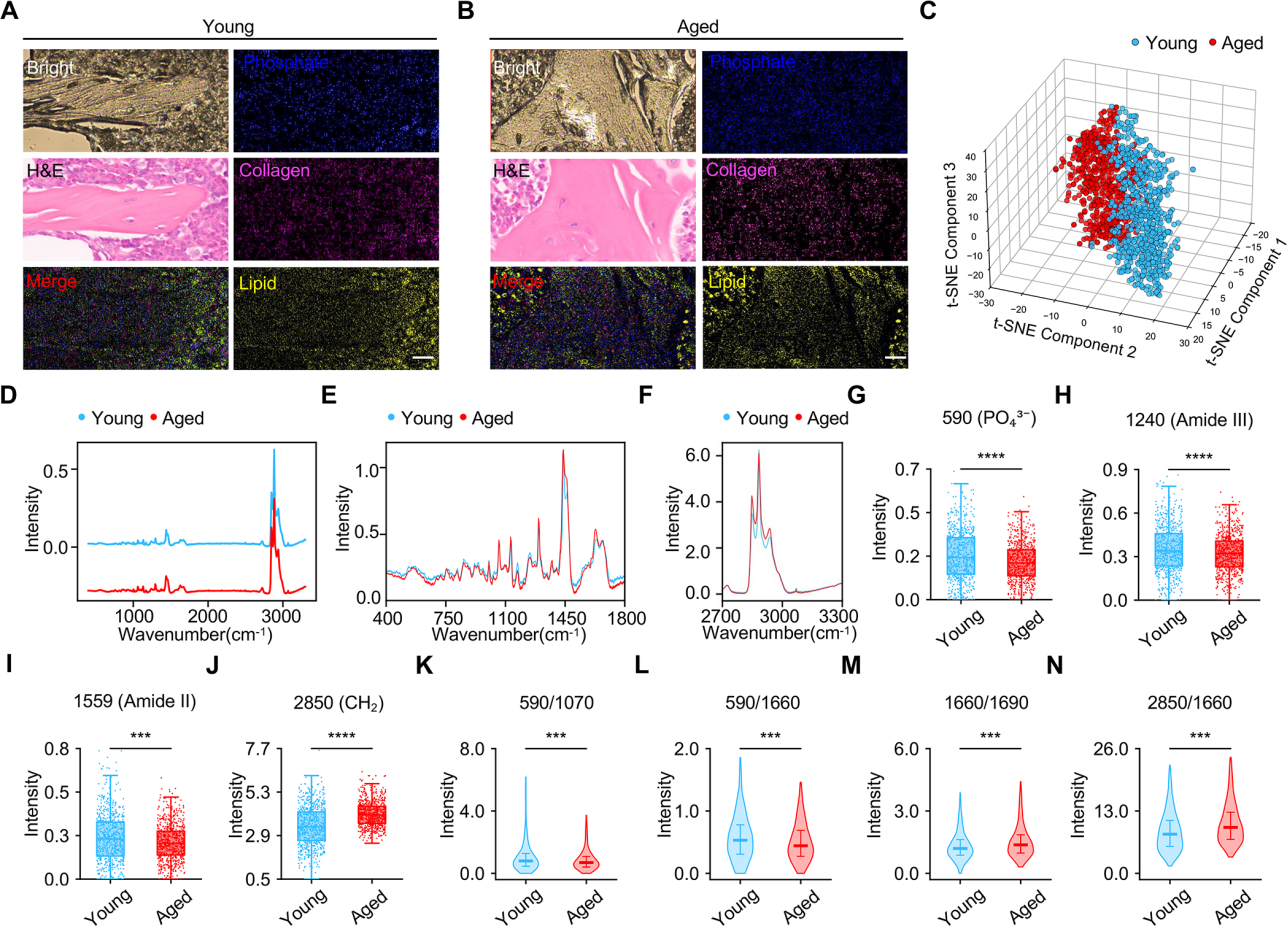
Confocal Raman imaging reveals a compositional fingerprint in young versus aged trabecular bone. **(A, B)** Representative bright-field images, H&E-stained sections, and confocal Raman maps of femoral trabecular bone from young and aged mice. Raman maps show the spatial distribution of mineral (PO_4_^3-^, 590 cm^-1^), collagen/matrix (Amide II, 1559 cm^-1^), and lipid (CH_2_, 2850 cm^-1^) signals. Scale bar, 2 μm. **(C)** 3D t-SNE visualization of normalized full-spectrum Raman datasets from trabecular bone in young and aged groups. **(D)** Representative Raman spectra extracted from trabecular regions (n = 6). **(E, F)** Group-averaged Raman spectra spanning the fingerprint window from 400 to 1800 cm^-1^ and the high-wavenumber window from 2700 to 3300 cm^-1^. **(G–J)** Quantification of peak intensities for phosphate (590 cm^-1^), collagen-related bands (Amide III, 1240 cm^-1^; Amide II, 1559 cm^-1^), and lipid CH_2_ stretching (2850 cm^-1^). **(K–N)** Raman-derived indices, including phosphate-to-carbonate (590 cm^-1^/1070 cm^-1^), phosphate-to-matrix (590 cm^-1^/1660 cm^-1^), collagen maturity (1660 cm^-1^/1690 cm^-1^), and lipid-to-matrix (2850 cm^-1^/1660 cm^-1^). Data are mean ± SD. ***P < 0.001, ****P < 0.0001.

To characterize osteoporosis-associated molecular remodeling directly within trabecular regions, we performed confocal Raman spectral imaging on femoral trabeculae and co-registered the mapped regions with H&E-stained sections. Raman imaging enabled *in situ* visualization of major bone constituents across trabecular structures and provided spatially resolved distributions of mineral-, matrix-, and lipid-associated signals within the same anatomical ROI (**Figures 1A, B**).

After background correction and area normalization, trabecular spectra were extracted for downstream analyses, and dimensionality reduction of the normalized full-spectrum data showed distinct group-wise distributions between the young and aged groups, indicating a robust population-level separation encoded in Raman fingerprints (**Figure 1C**). Focusing on the biochemical fingerprint regions 400–1800 cm^-1^ and high-wavenumber regions 2700–3300 cm^-1^, we observed coordinated remodeling across mineral, collagen, and lipid features (**Figures 1D–F**), with osteoporotic trabeculae exhibiting an overall decrease in phosphate-related signals (590 cm^-1^) together with reductions in the phosphate-to-carbonate ratio (590 cm^-1^/1070 cm^-1^) and phosphate-to-matrix ratio (590 cm^-1^/1660 cm^-1^) (**Figures 1G, K, L**). In parallel, collagen-associated bands including Amide II (1559 cm^-1^) and Amide III (1240 cm^-1^) were globally reduced, whereas the Amide I-derived collagen maturity index (1660 cm^-1^/1690 cm^-1^) increased (**Figures 1H, I, M**). Conversely, lipid-associated CH_2_ stretching bands (2850 cm^-1^) and CH_3_ stretching bands (2960 cm^-1^), along with the lipid-to-matrix ratio (2850 cm^-1^/1660 cm^-1^), were elevated in osteoporotic trabeculae (**Figures 1J, N**), and additional representative peaks related to mineral, collagen, and lipid features are also shown (**Supplementary Figure S1A–J**).

### 3.2 Full-spectrum machine learning enables automated discrimination of trabecular spectral phenotypes

**Figure 2:**
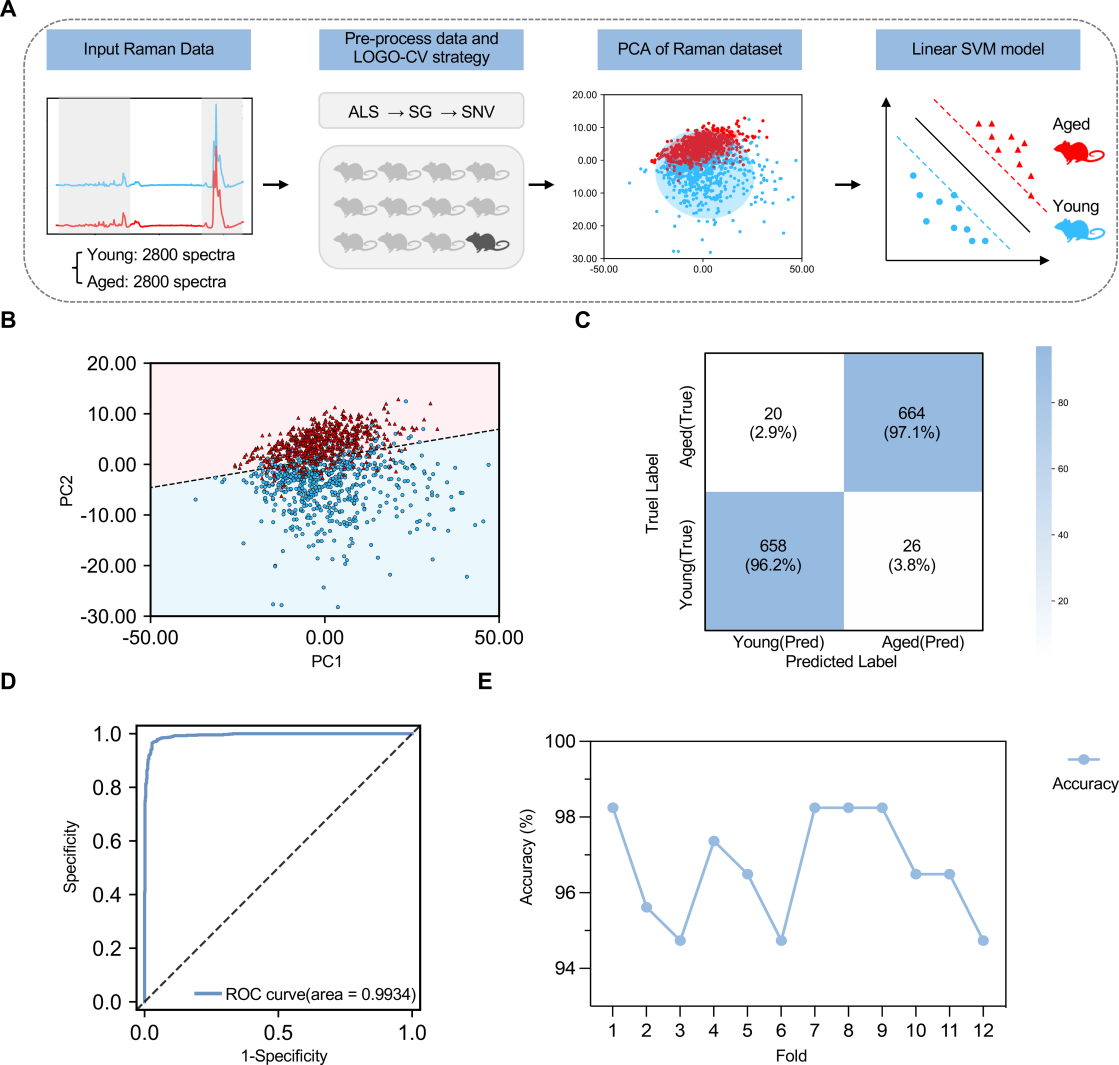
Full-spectrum machine learning pipeline for Raman-based age phenotyping of trabecular bone. **(A)** Workflow for Raman spectral preprocessing and linear support vector machine (LSVM) model construction using leave-one-group-out cross-validation (LOGO-CV). **(B)** Principal component analysis (PCA) projection of the Raman dataset with the LSVM decision boundary overlaid. **(C)** Confusion matrix summarizing LSVM classification performance for young versus aged trabecular spectra (percent, %). **(D)** Receiver operating characteristic (ROC) curve for the LSVM classifier. **(E)** Fold-wise classification accuracy across the 12 LOGO folds.

To evaluate whether Raman spectra can serve as quantitative phenotypes for automated age-stratification, we implemented a LSVM utilizing the high-dimensional trabecular Raman dataset (**Figure 2A**). We then visualized the spectral feature space using PCA together with the trained classifier, and the model defined a clear decision boundary in the reduced dimensional space that effectively segregated the young group from the aged group (**Figure 2B**), supporting the notion that age-related chemical remodeling carries sufficient discriminative information for classification. Model performance was quantified using leave-one-group-out cross-validation across 12 folds, and the resulting confusion matrix showed precise group segregation, correctly identifying 96.2% of spectra from the young group and 97.1% from the aged group (**Figure 2C**). ROC analysis further confirmed excellent discriminatory performance with an AUC of 0.9934 (**Figure 2D**). Consistent performance across 12 independent mice, reflected by a mean fold accuracy of 96.64 ± 1.38% (**Figure 2E**), underscores the ability of the model to extract robust biological signatures of aging. Collectively, these results demonstrate that trabecular Raman profiles contain stable biomolecular signatures that support high-fidelity phenotyping and robust classification.

### 3.3 Raman compositional remodeling covaries with trabecular microarchitecture and local mechanical properties

**Figure 3:**
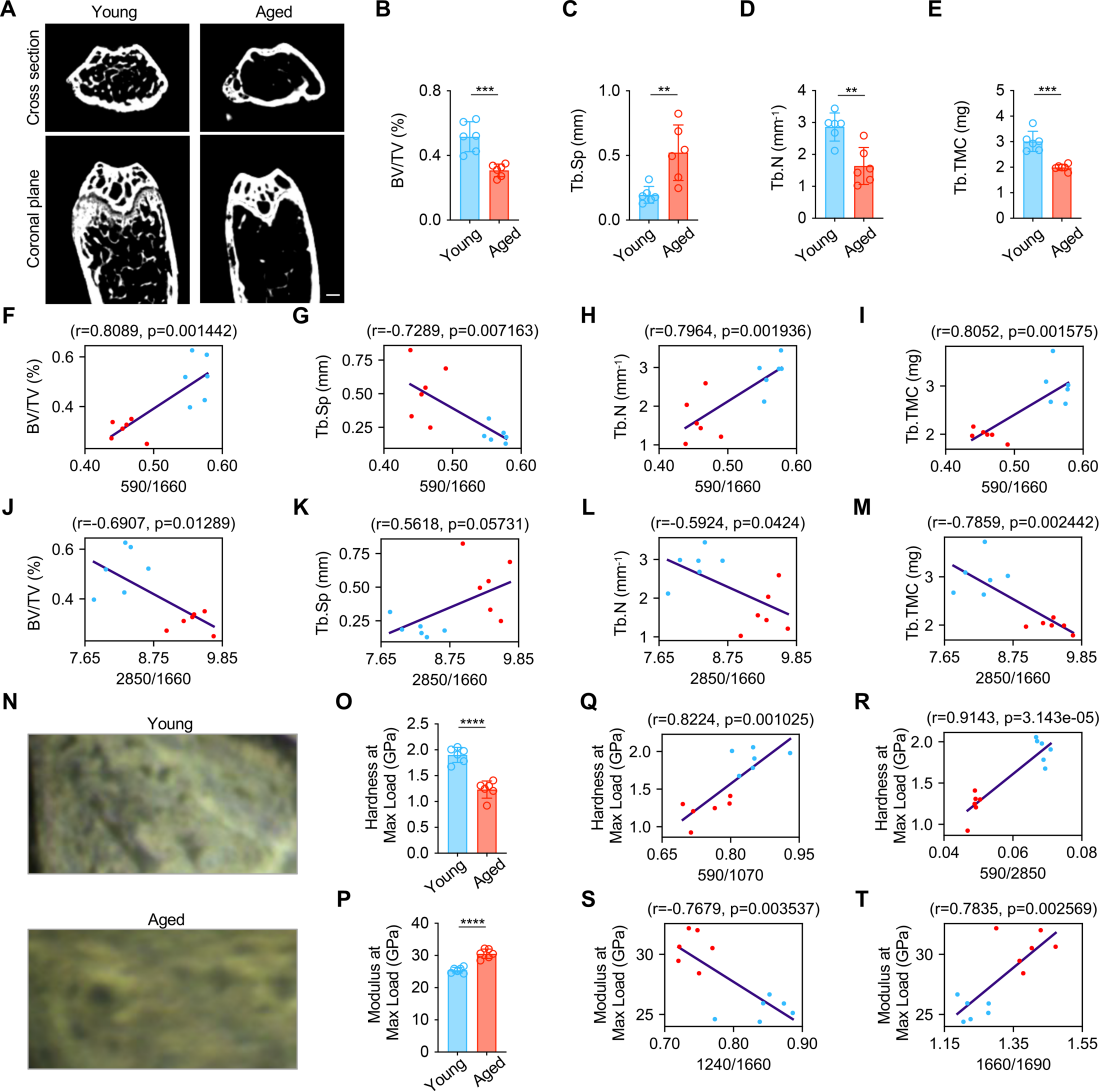
Associations between Raman compositional metrics, trabecular microarchitecture, and local mechanics in young versus aged mice. **(A)** Representative micro-CT reconstructions of femora from young and aged mice (coronal plane and cross section). Scale bar, 300 μm. **(B–E)** Quantification of trabecular bone morphometric parameters, including bone volume fraction (BV/TV), trabecular separation (Tb.Sp), trabecular number (Tb.N), and trabecular tissue mineral content (Tb.TMC) (n = 6). **(F–M)** Pearson correlations between Raman indices (phosphate-to-matrix, 590/1660 cm^-1^; lipid-to-matrix, 2850/1660 cm^-1^) and trabecular micro-CT parameters (BV/TV, Tb.Sp, Tb.N, Tb.TMC). **(N)** Representative nanoindentation images of trabecular regions. **(O, P)** Quantification of nanoindentation-derived hardness and elastic modulus in young and aged groups. **(Q–T)** Pearson correlations of hardness with phosphate-to-carbonate (590 cm^-1^/1070 cm^-1^) and phosphate-to-lipid (590 cm^-1^/2850 cm^-1^), and of elastic modulus with collagen disorder (1240/1660 cm^-1^) and collagen maturity (1660 cm^-1^/1690 cm^-1^). Data are mean ± SD. **P < 0.01, ***P < 0.001, ****P < 0.0001.

To determine whether Raman fingerprints reflect structural deterioration, we performed micro-CT of the distal femoral trabecular compartment, which revealed a more porous architecture and an enlarged marrow cavity in the aged group (**Figure 3A**). The aging-associated osteoporotic phenotype was confirmed by decreases in bone volume fraction (BV/TV), trabecular number (Tb.N), and trabecular tissue mineral content (TMC), together with an increase in trabecular separation (Tb.Sp) (**Figures 3B–E**). Concomitant decreases in trabecular thickness (Tb.Th), connectivity, and connectivity density (Conn.D) further supported this phenotype (**Supplementary Figure S2A–C**).

We next examined whether composition-sensitive Raman metrics tracked these architectural parameters. Pearson correlation analysis showed that Raman-derived ratios reflecting mineral-to-matrix balance (590 cm^-1^/1660 cm^-1^) and lipid-to-matrix balance (2850 cm^-1^/1660 cm^-1^) were significantly associated with trabecular micro-CT indices (**Figures 3F–M**; **Supplementary Figure S2D–I**), indicating that Raman fingerprints encode structure-relevant information rather than isolated spectral changes.

To link composition with local tissue-level mechanics, we performed nanoindentation within trabecular regions (**Figure 3N**). Trabecular bone from the aged group exhibited significantly reduced hardness, whereas elastic modulus increased (**Figures 3O, P**). Importantly, Raman parameters related to physicochemical bone quality, including the phosphate-to-carbonate ratio (590 cm^-1^/1070 cm^-1^), phosphate-to-lipid ratio (590 cm^-1^/2850 cm^-1^), collagen maturity (1660 cm^-1^/1690 cm^-1^), and collagen structural disorder (1240 cm^-1^/1660 cm^-1^), were significantly correlated with nanoindentation-derived mechanical metrics (**Figures 3Q–T**). These findings support Raman spectroscopy as an integrative readout that captures molecular composition together with microscale mechanical competence.

### 3.4 Single-cell transcriptomics reveals BMMSC program shifts that concord with Raman-defined fingerprints

**Figure 4:**
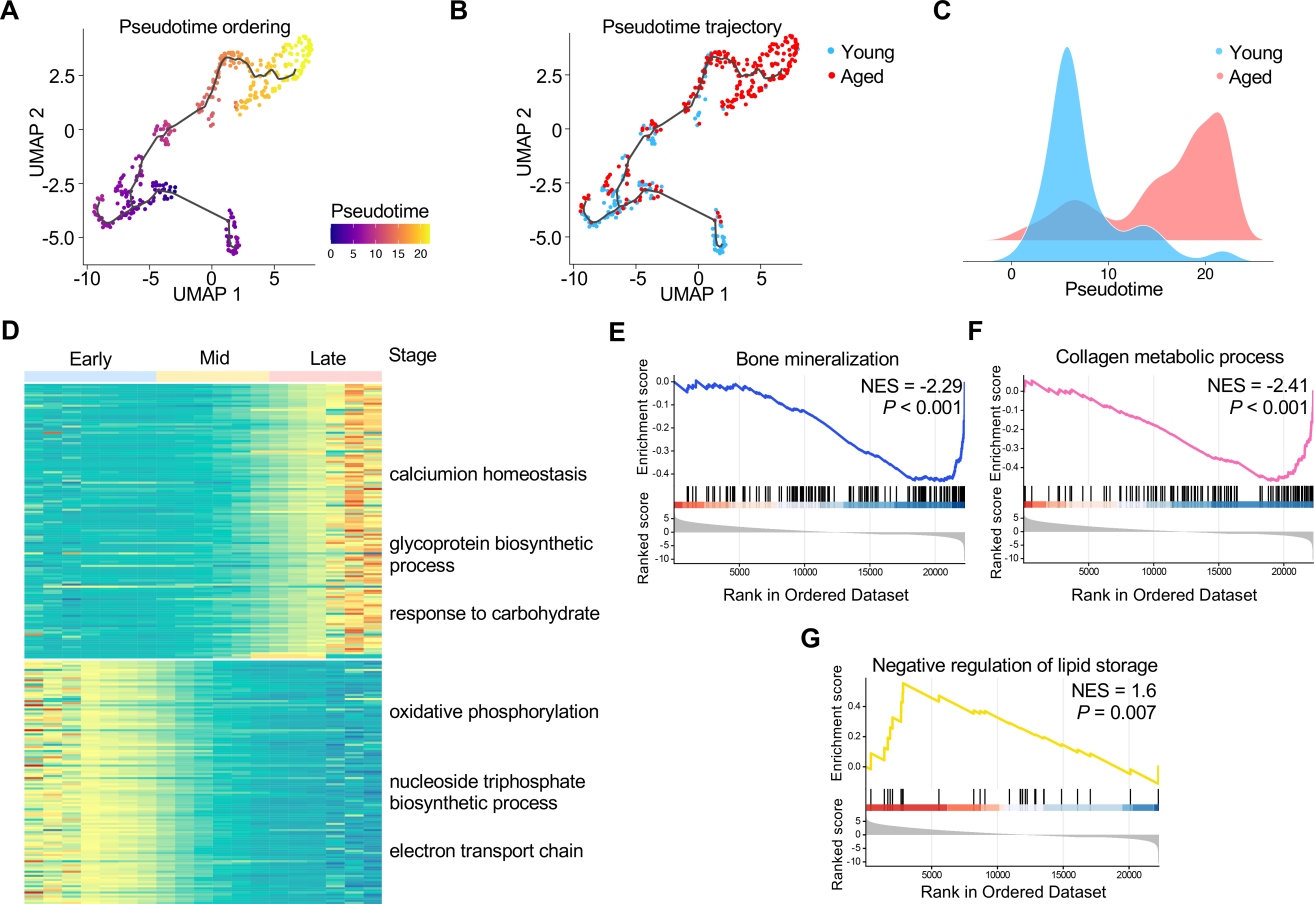
Single-cell sequencing reveals age-dependent divergence in BMMSC pseudotime trajectories and associated pathway-level remodeling. **(A)** UMAP embedding of BMMSCs colored by inferred pseudotime. **(B)** Distribution of young and aged cells along the pseudotime trajectory. **(C)** Density plot comparing pseudotime distributions between groups. **(D)** Pseudotime module heatmap of BMMSCs arranged by early, mid, and late stages. **(E–G)** Gene set enrichment analysis (GSEA) results for BMMSCs transcriptomes showing pathways related to bone mineralization, collagen metabolic process, and negative regulation of lipid storage.

To connect tissue compositional remodeling with the underlying cellular programs, we analyzed bone marrow scRNA-seq data and focused on BMMSCs after clustering and annotation (**Supplementary Figure S3A**). Pseudotime analysis revealed clear trajectory differences between the young and aged groups, and the module-gene pseudotime heatmap further demonstrated distinct temporal gene-expression patterns, indicating that transcriptional states alone could distinguish the two groups (**Figures 4A–D**). Pathway-level analyses showed coordinated remodeling across phosphate regulation, collagen metabolism, and lipid biology. Gene set enrichment analysis highlighted downregulation of mineralization- and collagen-related pathways together with downregulation of pathways involved in negative regulation of lipid processes (**Figures 4E–G**).

**Figure 5:**
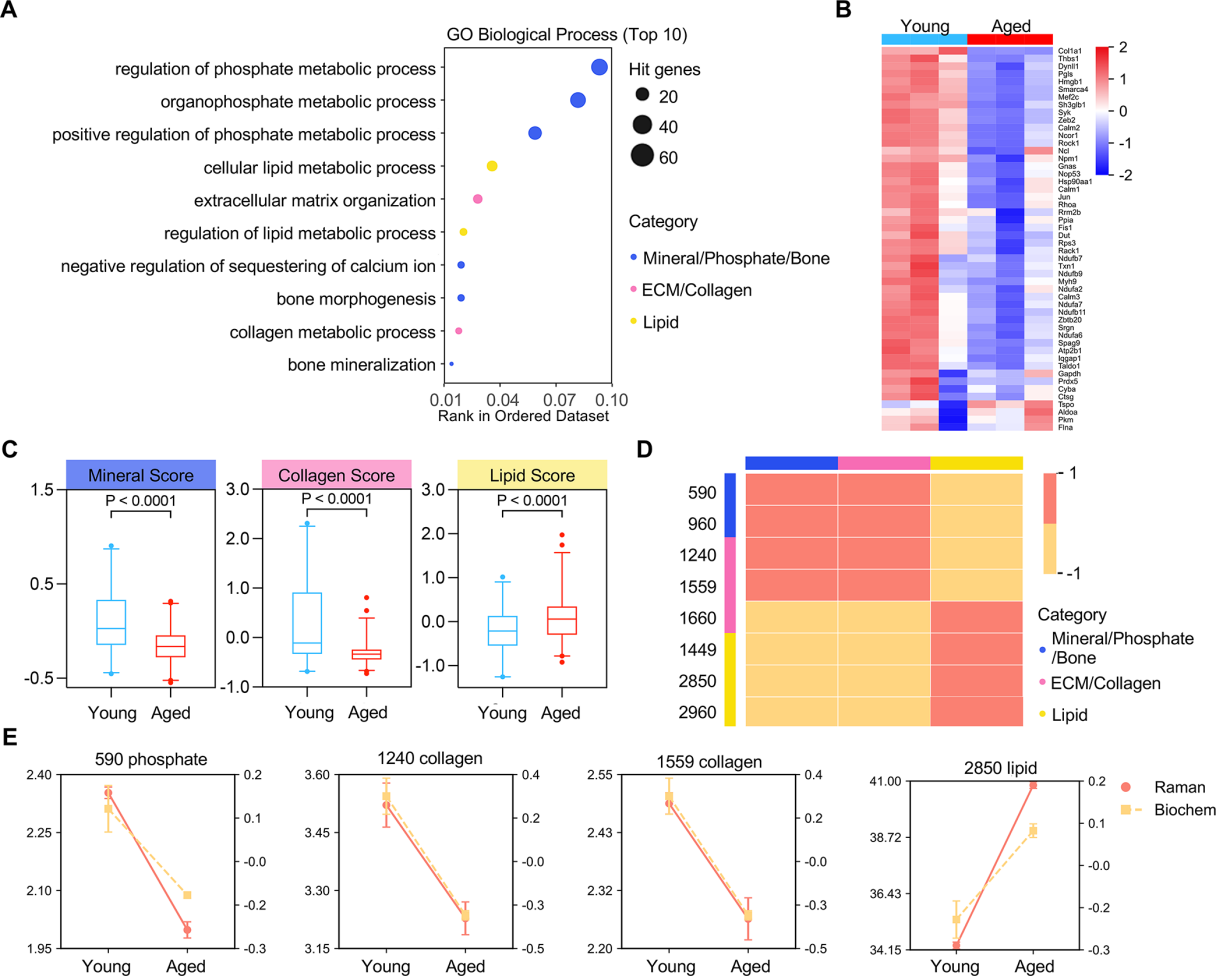
Single-cell sequencing–derived module scores show concordance with Raman spectral features. **(A)** GO BP enrichment for BMMSCs highlighting mineral/phosphate-, ECM/collagen-, and lipid-related pathways. **(B)** Heatmap of representative genes associated with these pathway categories. **(C)** Module scores for mineral, collagen, and lipid gene sets and comparison between young and aged groups. **(D)** Heatmap linking representative Raman peaks (mineral/phosphate, ECM/collagen, lipid) with corresponding gene modules to evaluate directional consistency. **(E)** Group comparisons of representative Raman peaks (590, 1240, 1559, 2850 cm^-1^) alongside the matched module trends. Data are mean ± SD.

Gene Ontology (GO) biological process (BP) enrichment focused on phosphate-, collagen-, and lipid-related terms recapitulated these directional trends (**Figures 5A, B**). To directly test whether Raman signatures mirrored these transcriptional modules, we constructed pathway gene modules for phosphate, collagen, and lipid processes and calculated module scores (**Figure 5C**). Concordance analyses comparing module scores with Raman peak intensities demonstrated clear consistency across all three axes, and concordance heatmaps captured the overall alignment between transcriptomic remodeling and Raman-defined compositional shifts (**Figures 5D, E**; **Supplementary Figure S3B**).

### 3.5 OVX-induced osteoporosis recapitulates the Raman fingerprint and its coupling to structure and mechanics

**Figure 6:**
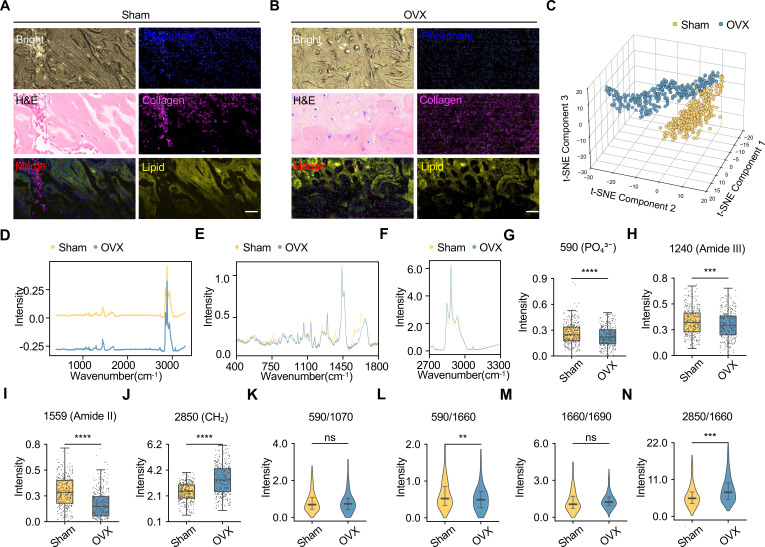
OVX-induced osteoporosis shows a Raman compositional pattern consistent with the aging model. **(A, B)** Representative bright-field images, H&E-stained sections, and confocal Raman maps of femoral trabecular bone from sham and OVX mice. Raman maps show mineral (PO_4_^3-^, 590 cm^-1^), collagen/matrix (Amide II, 1559 cm^-1^), and lipid (CH_2_, 2850 cm^-1^) signals. Scale bar, 2 μm. **(C)** 3D t-SNE visualization of normalized full-spectrum Raman datasets from sham and OVX trabecular bone. **(D)** Representative Raman spectra extracted from trabecular regions (n = 4). **(E, F)** Group-averaged Raman spectra covering 400–1800 cm^-1^ for the fingerprint window and 2700–3300 cm^-1^ for the high-wavenumber window. **(G–J)** Quantification of phosphate (590 cm^-1^), collagen-related bands (1240 and 1559 cm^-1^), and lipid CH_2_ stretching (2850 cm^-1^). **(K–N)** Raman-derived indices, including phosphate-to-carbonate (590 cm^-1^/1070 cm^-1^), phosphate-to-matrix (590 cm^-1^/1660 cm^-1^), collagen maturity (1660 cm^-1^/1690 cm^-1^), and lipid-to-matrix (2850 cm^-1^/1660 cm^-1^). Data are mean ± SD. ns, not significant. **P < 0.01, ***P < 0.001, ****P < 0.0001.

To further validate the relevance of Raman spectroscopy in osteoporosis, we established an OVX mouse model. We analyzed femoral trabeculae from the sham and OVX groups by H&E staining and Raman spectroscopy, which clearly resolved the major components of trabecular bone (**Figures 6A, B**). Dimensionality reduction revealed distinct separation between the two groups, and this separation was more pronounced than that observed between the young and aged groups, suggesting that OVX elicited stronger compositional remodeling within the analyzed timeframe (**Figure 6C**). The spectral distribution characteristics in the Raman fingerprint region of the OVX model were broadly comparable to those of the aged group (**Figures 6D–F**). Analysis of the Raman fingerprint region further showed that the overall spectral changes were consistent with aging-associated osteoporosis, with only subtle differences in a subset of indices. Notably, no significant differences were detected in the collagen-related peak at 1660 cm^-1^, the collagen maturity ratio (1660 cm^-1^/1690 cm^-1^), or the phosphate-to-carbonate ratio (**Figures 6G–N**; **Supplementary Figure S4A–J**).

**Figure 7:**
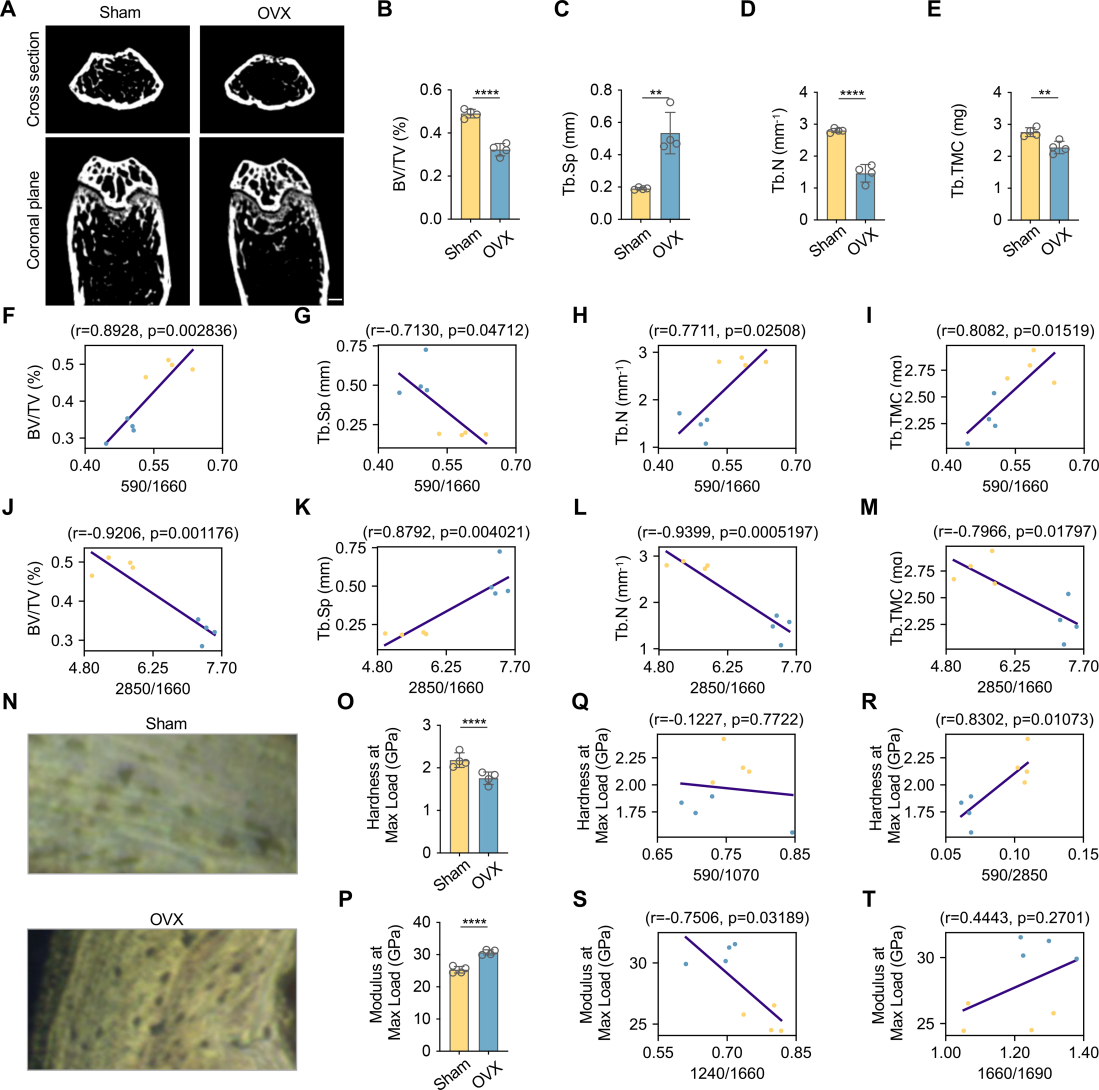
OVX model validates the coupling of Raman features with trabecular microarchitecture and nanoindentation outcomes. **(A)** Representative micro-CT images of femurs from sham and OVX mice. **(B–E)** Quantification of trabecular parameters including BV/TV, Tb.Sp, Tb.N, and Tb.TMC in sham and OVX groups (n = 4). Scale bar, 300 μm. **(F–M)** Pearson correlation analyses between phosphate-to-matrix ratio (590 cm^-1^/1660 cm^-1^) or lipid-to-matrix ratio (2850 cm^-1^/1660 cm^-1^) derived from Raman spectra and the corresponding micro-CT trabecular parameters. **(N)** Representative nanoindentation images of trabecular bone. **(O, P)** Quantification of hardness and elastic modulus in sham and OVX groups. **(Q–T)** Pearson correlation analyses of hardness with Raman phosphate-to-carbonate ratio (590 cm^-1^/1070 cm^-1^) and phosphate-to-lipid ratio (590 cm^-1^/2850 cm^-1^), and of elastic modulus with collagen disorder index (1240 cm^-1^/1660 cm^-1^) and collagen maturity ratio (1660 cm^-1^/1690 cm^-1^). Data are mean ± SD. **P < 0.01, ****P < 0.0001.

To test reproducibility across etiologically distinct osteoporosis contexts, we applied the same multimodal pipeline to the OVX model. Micro-CT confirmed a robust osteoporotic phenotype after OVX (**Figure 7A**), including decreased BV/TV, Tb.N, and trabecular TMC together with increased Tb.Sp (**Figures 7B–E**), whereas the trends in Tb.Th, connectivity, and Conn.D were similar to those observed in the aged model (**Supplementary Figure S5A–C**). Raman-to-structure comparisons showed that Raman readouts continued to reflect trabecular architectural deterioration effectively in OVX animals (**Figures 7F–M**; **Supplementary Figure S5D–I)**. Moreover, nanoindentation results were consistent with the aging-associated osteoporosis phenotype, showing significantly reduced hardness together with increased elastic modulus (**Figures 7N–P**). Raman compositional features again exhibited significant correlations with mechanical parameters, indicating that the structure–mechanics–composition coupling captured by Raman is reproducible across models (**Figures 7Q–T**).

## 4 Discussion

Raman spectroscopy has long been used to assess bone-matrix composition through established metrics, including mineral-to-matrix ratio, carbonate substitution, crystallinity, and collagen-related sub-peak features (12). Methodological reviews have highlighted its biochemical sensitivity while also underscoring the importance of standardized preprocessing for reproducibility (13). Translational studies further suggest that Raman-derived matrix properties capture aspects of bone quality that are not reflected by mineral density alone (14, 15). Although Raman spectroscopy has been applied to evaluate bone status in a variety of settings, relatively few studies have examined its potential specifically in osteoporosis (16). In the present study, we integrated Raman compositional profiling with microarchitectural imaging, local mechanical testing, and single-cell transcriptomics, demonstrating that Raman spectroscopy can serve as a rapid, multidimensional readout linking chemical composition to structural, mechanical, and cellular alterations. This multi-scale framework underscores the unique value of Raman-based assessment in capturing coordinated osteoporosis-related changes across biological and functional levels. Importantly, our findings indicate that Raman-derived signals can reflect multidimensional aspects of bone quality and may support the future development of comprehensive Raman-based evaluation strategies for osteoporosis.

The aging-associated reduction in phosphate-related Raman features is consistent with altered mineralization status and mineral chemistry, whereas the concurrent decline in matrix-associated bands indicates remodeling of the collagen-rich extracellular matrix (17). Both trends are in line with previous work establishing Raman spectroscopy as a sensitive reporter of coupled inorganic and organic matrix changes in bone (18, 19). Notably, despite reduced bulk collagen signals across major trabecular regions, the Amide I-derived collagen maturity index increased, suggesting a shift toward a more mature and highly cross-linked network. This pattern is consistent with age-associated reductions in collagen remodeling accompanied by increased cross-link formation and elevated levels of advanced glycation end products (20), and it provides a plausible explanation for the nanoindentation findings of reduced hardness but increased elastic modulus in both aging and OVX models. Increased collagen maturity may enhance fibrillar load transfer and increase modulus while simultaneously limiting molecular sliding and post-yield deformation, thereby lowering hardness (21). At the same time, reduced phosphate-related mineral signals together with increased lipid-associated features may further compromise resistance to plastic deformation by perturbing local composite balance, hydration, and susceptibility to microdamage, even when elastic stiffness is elevated. Against a background of pronounced microarchitectural deterioration, a higher tissue-level modulus is therefore unlikely to translate into improved whole-bone performance and may instead reflect greater fragility arising from a stiffer, more mature collagen matrix embedded within a compromised architecture. Consistent with this framework, lipid-associated CH_2_/CH_3_ stretching signals increased in osteoporotic trabecular regions. Although these bands are not specific to individual lipid species, the direction of change matches the well-described age-related shift of the marrow niche toward adipogenesis at the expense of osteogenesis (22, 23).

Given their multipotency and central roles in bone formation and niche regulation, MSC transcriptional remodeling represents an important molecular correlate of osteoporotic change (24). Our single-cell transcriptomic analysis provides a molecular anchor for the Raman mineral–collagen–lipid fingerprint by revealing coordinated pathway-level reprogramming within BMMSCs, in which mineralization- and collagen-related module scores decreased whereas lipid-associated programs increased, mirroring the directionality of the Raman shifts observed in trabecular bone (25–27). This transcriptome–composition concordance supports the interpretation that the Raman phenotype reflects an integrated readout of BMMSC state transitions and downstream tissue remodeling rather than isolated changes in individual peak ratios. Because Raman spectra are intrinsically high-dimensional and encode correlated variation across mineral, collagen, and lipid regions, they are well suited to full-spectrum multivariate modeling that can capture subtle multi-band patterns beyond manually derived metrics (28–30). In this context, LSVM-based discrimination of trabecular spectra from young and aged mice demonstrates the feasibility of automated Raman phenotyping in bone and offers a computational route for translating complex spectral fingerprints into quantitative, disease-relevant readouts that are consistent with BMMSC program shifts (31). Although Raman spectroscopy does not directly measure gene expression, convergence between scRNA-seq module trends and spectral features supports the plausibility that BMMSC reprogramming is accompanied by compositional signatures that are detectable and classifiable at the tissue level. Future studies should prioritize cross-instrument and cross-cohort validation, together with harmonized preprocessing and feature selection, to improve model transferability; calibration or domain-adaptation strategies may further facilitate robust deployment of label-free Raman phenotyping across acquisition settings.

Although natural aging and OVX both lead to trabecular bone loss, they represent distinct pathogenic trajectories (32, 33). Aging integrates chronic MSC senescence, oxidative and glycation stress, and a progressive decline in matrix turnover, whereas OVX primarily models abrupt estrogen withdrawal with a rapid, high-turnover imbalance that is strongly trabecula-biased and time-dependent (34, 35). Accordingly, the shared Raman fingerprint of reduced phosphate- and matrix-related signals together with elevated lipid-associated features suggests a convergent mineral–matrix–marrow remodeling axis, even though selected metrics may diverge between models. In our OVX cohort, the phosphate-to-carbonate ratio (590 cm^-1^/1070 cm^-1^) and the Amide I band (1660 cm^-1^) were not significantly altered, which may reflect limited time for secondary mineralization and collagen cross-link accumulation, averaging of newly formed and pre-existing tissue during accelerated remodeling, and the possibility that estrogen deficiency perturbs hydration and mineral heterogeneity before producing the longer-term collagen maturity changes typical of aging. Despite these model-specific differences, OVX reproduced both the direction of change and the structure–mechanics coupling of the core Raman phenotype, supporting the robustness and reproducibility of this compositional fingerprint across etiologically distinct osteoporotic contexts.

Several limitations should be acknowledged. First, the Raman data were acquired from prepared bone sections, and sample preparation may influence absolute intensity and background fluorescence, underscoring the need for harmonized protocols and inter-study calibration. Although decalcification may affect absolute Raman signal intensities, identical processing conditions minimize relative signal bias, and future work will further optimize sample preparation to minimize its impact on the results. Second, although correlations between Raman metrics and mechanical properties support functional relevance, they do not establish causality; targeted interventions that modulate mineralization, collagen cross-linking, or marrow adiposity would help clarify the causal drivers of the Raman signature. Third, our single-cell transcriptomic analysis focused on BMMSCs, as they are most directly involved in the synthesis and regulation of bone mineral, collagen, and lipid components; nevertheless, other cell populations potentially related to bone metabolism, such as monocytes or macrophages, were not systematically analyzed, and contributions from bone-surface or matrix-embedded cells may therefore not be fully captured, suggesting that improved anatomical alignment could be achieved by integrating spatial transcriptomics or multiplexed imaging. Nevertheless, detecting osteoporotic remodeling with composition-sensitive techniques remains highly attractive because current practice relies predominantly on structural and density surrogates, and progress toward *in vivo* Raman assessment of bone is increasingly feasible (36, 37). Probe-based Raman geometries have been validated on human bone specimens for matrix-quality evaluation, and spatially offset Raman spectroscopy has enabled longitudinal analysis of tibial bone aging in mice, both transcutaneously and on exposed bone, indicating the potential for non-destructive monitoring through overlying tissue (38, 39). Together with advances in probe-based or spatially offset Raman technologies and multivariate data analysis, these findings support the future use of Raman-derived compositional fingerprints as rapid and comprehensive indicators of tissue-level bone quality, which may complement BMD, high-resolution peripheral quantitative computed tomography (HR-pQCT), and clinical risk models to improve osteoporosis detection and fracture-risk stratification (40, 41).

## 5 Conclusion

**Figure 8:**
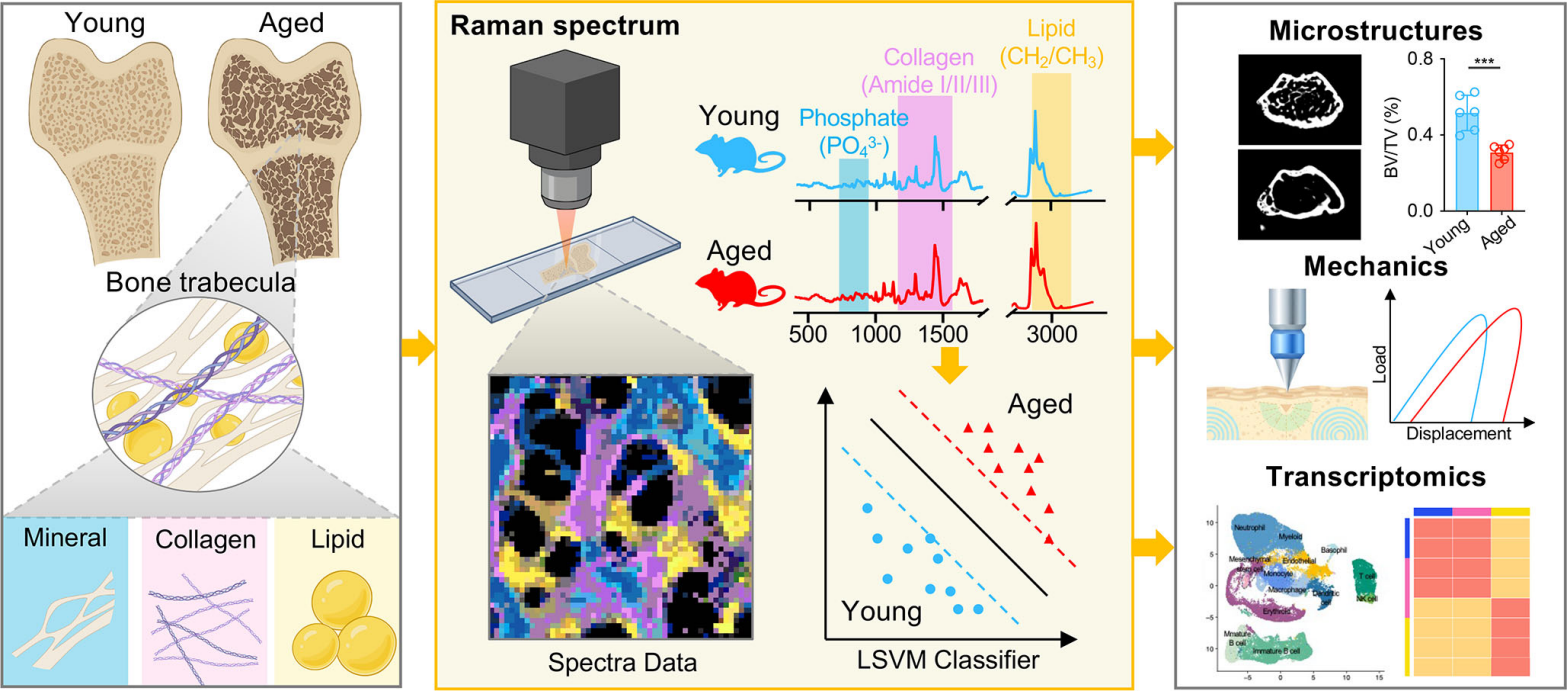
Graphical summary of the study design and principal findings. Raman-based compositional fingerprints revealed osteoporosis-related alterations in trabecular bone and their associations with microstructural, mechanical, and transcriptomic features.

In this study, we established an integrated multimodal framework to interrogate trabecular bone fragility in osteoporosis models. We identified a conserved Raman spectral fingerprint characterized by reduced mineral and matrix signals together with increased lipid contributions, and showed that this fingerprint tracked microstructural deterioration, local mechanical alterations manifested by reduced hardness and increased elastic modulus, and aging-associated transcriptional reprogramming of phosphate-, collagen-, and lipid-related pathways in BMMSCs. Taken together, these findings support Raman spectroscopy as a multidimensional compositional readout that can be integrated with microarchitectural imaging, mechanical testing, and single-cell transcriptomics, thereby providing mechanistic insight beyond conventional metrics and supporting the development of translational, potentially noninvasive diagnostic strategies for osteoporosis (**Figure 8**).

## Data Availability

The raw data supporting the conclusions of this article will be made available by the authors, without undue reservation.
