# Supplementary material for: Raman spectroscopic fingerprinting uncovers a multi-scale structural–mechanical–transcriptomic coupling landscape in osteoporosis
Source: Front Endocrinol (Lausanne). 2026 May 28;17:1860651. doi: 10.3389/fendo.2026.1860651 (PMC13253466; doi:10.3389/fendo.2026.1860651)
Supplement: Supplementary file 1 [file Table1.docx]

Supplementary Material

**Figure S1. Confocal Raman imaging reveals a compositional fingerprint in young versus aged trabecular bone**

(A-E) Quantification of peak intensities for phosphate (960 cm⁻¹), carbonate (1070 cm⁻¹), lipid CH_2_ stretching (1449 cm⁻¹), collagen-related bands (Amide I, 1660 cm⁻¹), and lipid CH_3_ stretching (2960 cm⁻¹). (F-J) Raman-derived indices, including collagen maturity (880 cm⁻¹/860 cm⁻¹), phosphate-to-carbonate (960 cm⁻¹/1070 cm⁻¹), phosphate-to-matrix (960 cm⁻¹/1660 cm⁻¹) and lipid-to-matrix (1449 cm⁻¹/1660 cm⁻¹ and 2960 cm⁻¹/1660 cm⁻¹). Data are mean ± SD. ns, not significant. **P* < 0.05, ***P* < 0.01, ****P* < 0.001, *****P* < 0.0001.

**Figure S2. Associations between Raman compositional metrics and trabecular microarchitecture in young versus aged mice**

(A-C) Quantification of trabecular bone morphometric parameters, including trabecular thickness (Tb.Th), connectivity, and connectivity density (Conn.D) (n = 6). (D-I) Pearson correlations between Raman indices (phosphate-to-matrix, 590 cm⁻¹/1660 cm⁻¹; lipid-to-matrix, 2850 cm⁻¹/1660 cm⁻¹) and trabecular micro-CT parameters (Tb.Th, connectivity, Conn.D). Data are mean ± SD. ns, not significant. ***P* < 0.01, ****P* < 0.001.

**Figure S3. Single-cell transcriptomic programs in BMMSCs align with Raman-derived compositional axes**

(A) UMAP embedding of bone marrow single-cell RNA-seq data showing annotated major cell populations. (B) Concordance plot comparing group-wise changes between young and aged groups for Raman compositional features and corresponding transcriptomic module trends, including phosphate (960 cm⁻¹), lipid (1449 and 2960 cm⁻¹) and collagen (1660 cm⁻¹). Data are mean ± SD.

**Figure S4. OVX-induced osteoporosis shows a Raman compositional pattern consistent with the aging model**

(A-E) Quantification of peak intensities for phosphate (960 cm⁻¹), carbonate (1070 cm⁻¹), lipid CH_2_ stretching (1449 cm⁻¹), collagen-related bands (Amide I, 1660 cm⁻¹), and lipid CH_3_ stretching (2960 cm⁻¹). (F-J) Raman-derived indices, including collagen maturity (880 cm⁻¹/1860 cm⁻¹), phosphate-to-carbonate (960 cm⁻¹/1070 cm⁻¹), phosphate-to-matrix (960 cm⁻¹/1660 cm⁻¹) and lipid-to-matrix (1449 cm⁻¹/1660 cm⁻¹ and 2960 cm⁻¹/1660 cm⁻¹). Data are mean ± SD. ns, not significant. **P* < 0.05, ***P* < 0.01, ****P* < 0.001, *****P* < 0.0001.

**Figure S5. OVX model validates the coupling of Raman features with trabecular microarchitecture**

(A-C) Quantification of trabecular bone morphometric parameters, including trabecular thickness (Tb.Th), connectivity, and connectivity density (Conn.D) (n = 4). (D-I) Pearson correlations between Raman indices (phosphate-to-matrix, 590 cm⁻¹/1660 cm⁻¹; lipid-to-matrix, 2850 cm⁻¹/1660 cm⁻¹) and trabecular micro-CT parameters (Tb.Th, connectivity, Conn.D). Data are mean ± SD. ns, not significant. ***P* < 0.01, ****P* < 0.001.
